# Current status of mathematical foundations of the Gestalt concepts in psychophysics

**DOI:** 10.1167/jov.26.2.10

**Published:** 2026-02-18

**Authors:** Zygmunt Pizlo, Jie Z. Wang, Barbara Dosher, Robert M. Steinman

**Affiliations:** 1 Department of Cognitive Sciences, University of California, Irvine, CA, USA; 2 Center for Visual Science, Department of Brain and Cognitive Sciences, University of Rochester, Rochester, NY, USA; 3 Department of Psychology, University of Maryland, College Park, MD, USA

**Keywords:** psychophysics, simplicity, least-action, invariance, gestalt

## Abstract

Eileen Kowler was a member of the team of cognitive scientists that was assembled over a half a century ago by Robert M. Steinman. This team had grown and spanned several institutions in this country, as well as in Europe. The mechanisms governing the movements of the eyes were always at the center of Eileen's attention, but her research, as well as the research of our interdisciplinary group, is best understood by realizing its roots in Gestalt psychology. In the first part, this paper describes perspectives of two of the authors from the point of view of Eileen Kowler's collaborators. The second part provides a new look at the origins of Gestalt psychology, pointing out the scientific context of what has been referred to as the Gestalt revolution. The Gestalt revolution closely followed similar revolutions in mathematics and in physics, and it gave rise to new questions and challenges that have received recognition only recently.

## Introduction

Eileen received her graduate training with Robert Steinman, who studied with and was influenced by Hans Wallach and Jack Nachmias, Steinman's doctoral advisor. Steinman was directly influenced by other Gestalt researchers, including the Gleitmans and Mary Henle. Azriel Rosenfeld, a mathematician, computer vision researcher and a close colleague of Steinman at the University of Maryland in College Park, was also influenced by Gestalt ideas. Rosenfeld, together with Kowler and Steinman shaped the views and research of Pizlo when he moved to the United States. When Pizlo was starting his scientific career in Poland, he met Eileen Kowler at the Third European Conference on Eye Movements (ECEM 3) in Dourdan, France, in 1985. They met at Pizlo's poster that described his early work on symmetry and eye fixations. Meeting in a poster session was not common back then, because nobody attended poster sessions in ECEM conferences, including the authors of the posters. Poster sessions were effectively breaks for beer and wine. Pizlo's poster was an exception at that conference in 1985: Pizlo was personally present when Kowler brought Steinman to examine the poster. This encounter was shortly followed by extracting Pizlo from Poland and bringing him to the University of Maryland as a graduate student where he earned his second Ph.D., this one in psychology. His first Ph.D. was in Electronic Engineering. Kowler was instrumental in planning and pulling off this maneuver, and she became an informal advisor of Pizlo's doctoral work at the University of Maryland. This diverse group consisting of Pizlo, Kowler, and Steinman that became a group under such atypical conditions has stayed in close contact for more than 40 years. We had all assumed that Steinman, who is now 98, would be the one to die first (pass is often used today). This was not to be, so we decided to prepare a paper that would represent the kinds of problems our group liked to consider as we tried to build and maintain careers in academic science.

Steinman's interest in eye-movement research started at his graduate school, the New School for Social Research in New York City. Steinman, who began to practice dentistry in 1948, attended evening courses at the New School, and when he finished the coursework and exams required for a Ph.D. degree he went off, at Professor Hans Wallach's suggestion, to work with a young Assistant Professor Jacob (Jack) Nachmias, who had just moved from Psychology at Swarthmore College to Psychology at the University of Pennsylvania. Steinman closed his practice and moved to Philadelphia in 1958 to work on his Ph.D. under Nachmias’ supervision (Nachmias was the man in eye movements to know at the time because he had published two important eye movement papers in the *Journal of the Optical Society*, Nachmias, 1959; Nachmias, 1961). Steinman earned his Ph.D. in 1964 and accepted a job as a new Assistant Professor in Psychology at the University of Maryland in 1964. Apparently, he had some talent for politicking in the academy because by 1972 he was a Full Professor, probably because he had been invited to submit a lead article entitled “Miniature Eye Movement” to the journal *Science* (Steinman, Haddad, Skavenski, & Wyman, 1973). Steinman devoted most of professional career to making the best of all possible eye movement monitors, and one of the best was in place by 1980 shortly before the start of the interval of primary interest to readers of this paper. One also should note that Eileen Kowler joined Steinman's eye movement lab when she was 20 years old in 1972. She contributed a great deal to science in her lab, as well as in Steinman's lab, in the ensuing years. Steinman's activities beginning in 1985 cover a period that started with him working near and around his best efforts in science. Most of his important work during that period was collaborative, often with scientists from the Netherlands. Three Dutchmen stand out: Han Collewijn, and two of his postdocs, Casper Erkelens and Hans van der Steen. This team published the first accurate measurements of human vergence using the new Maryland Revolving Field Monitor (Erkelens, Steinman, & Collewijn, 1989; Erkelens, Van der Steen, Steinman, & Collewijn, 1989).

Below, the reader will find the perspectives of Jie Wang, Barbara Dosher, and Zygmunt Pizlo. Pizlo's perspective focuses on a new interpretation of the connection between Gestalt psychology and contemporary mathematics and physics.

## Jie Wang's perspective

**Figure 1.**
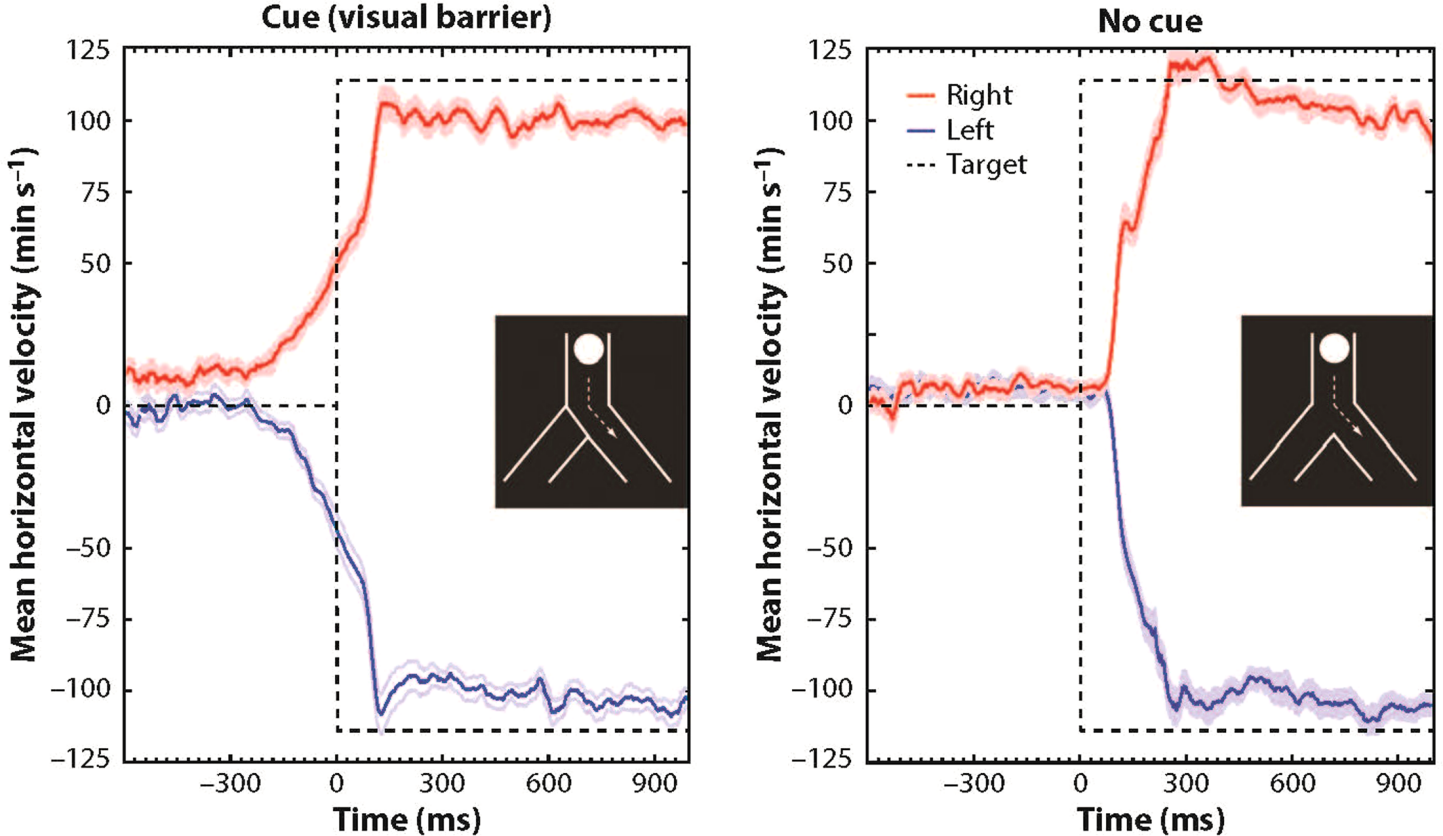
Visual geometrical cues can elicit ASEMs. (Left) When participants were instructed to pay attention to a target that moved along one of the oblique paths of an inverted Y-shaped stimulus, they consistently initiated ASEM responses (mean of horizontal eye velocity across subjects; error bar indicates ±1 *SE*) in the same direction of the unblocked path (red trace, right path; blue trace, left path) prior to time 0, which marks the onset of the horizontal target motion. (Right) When both oblique paths were open (i.e., no cue was present to indicate the future target path), ASEMs were absent in the average response and typical pursuit latency was observed. Adapted from Kowler et al. (2019), with permission.

**Figure 2.**
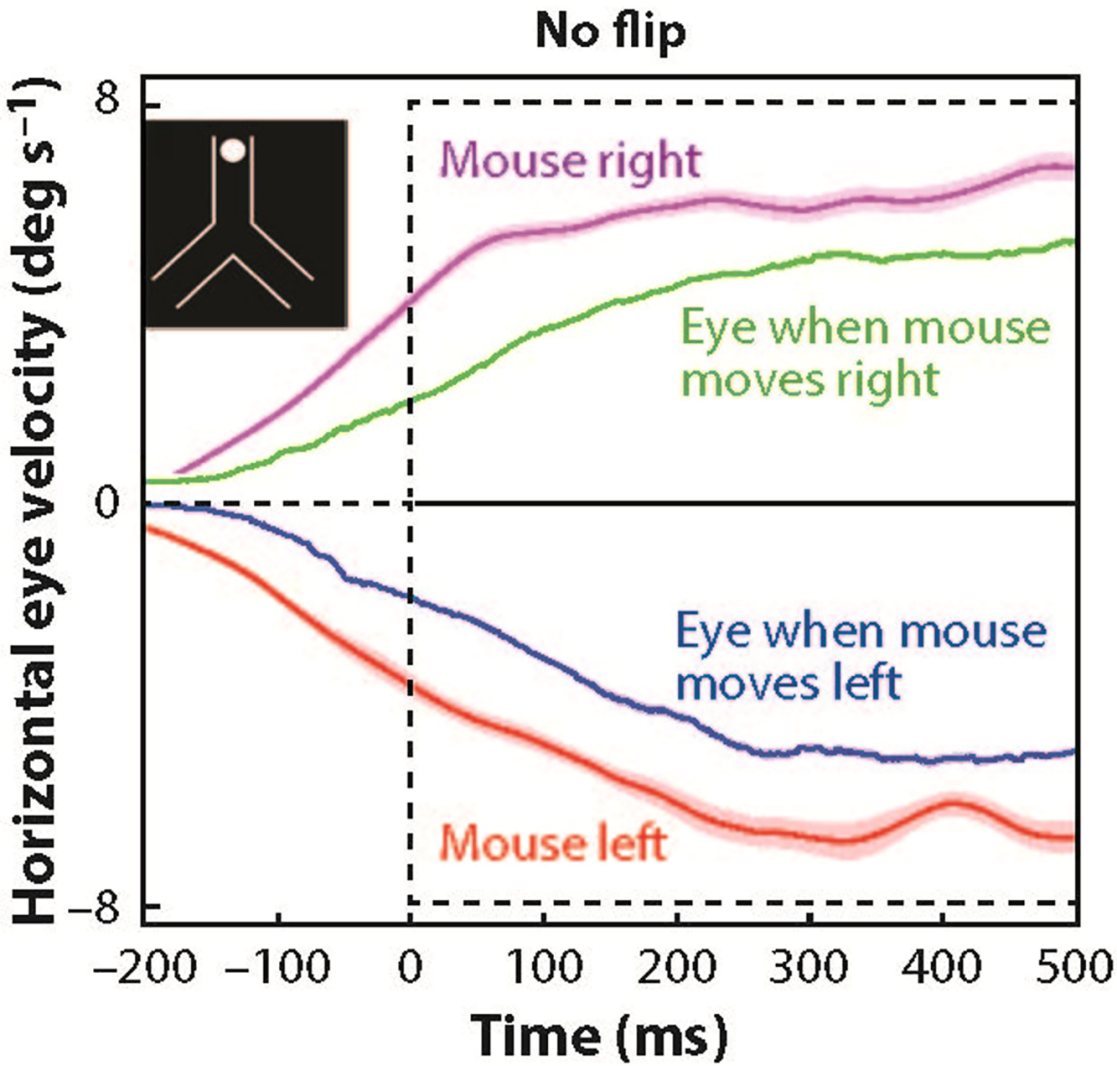
ASEMs emerge during self-produced target motion. Using the same inverted Y-shaped stimulus as in Figure 1, subjects freely chose either the left or right path and moved the target in the chosen path using a computer mouse. Self-produced target motions (“mouse right” or “mouse left”) effectively elicited corresponding ASEM responses in smooth-pursuit eye movements (“eye when mouse moves right” or “eye when mouse moves left”) with minimal temporal lag. Adapted from Kowler et al. (2019), with permission.

Eileen Kowler devoted more than four decades of her professional career to studying the control of human eye movements. She revolutionized the field by uncovering how smooth-pursuit eye movements rely on higher-level processes for motion prediction. Through her pioneering Y-shaped tube experiments, Kowler and students demonstrated that the anticipatory smooth eye movements (ASEMs), an indicator of predictive capability, can be elicited by memory and perceptual cues present in the scene (Kowler, 1989; Kowler, Aitkin, Ross, Santos, & Zhao, 2014; Santos & Kowler, 2017). For example, Figure 1 depicts the elicited ASEM response in the presence of a visual cue, in contrast to a case where cues were absent. Motor intentions can also be a contributing factor supporting prediction in smooth pursuit, as ASEM responses arise when subjects pursued a disc whose motion they controlled using a computer mouse (see Figure 2).

**Figure 3.**
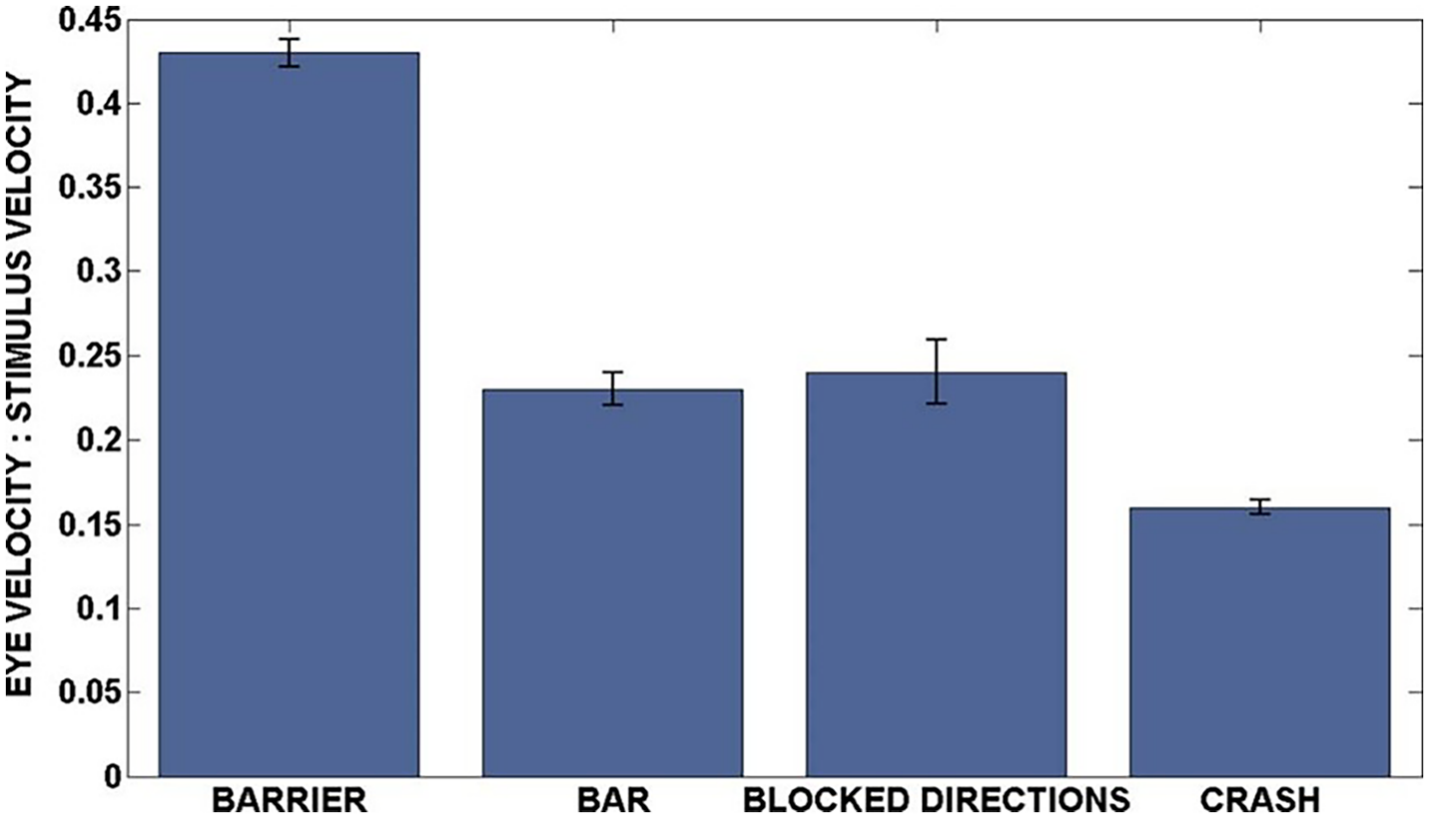
The strength of ASEM responses varies with cue type. ASEM responses were found in four conditions using the inverted Y-shaped stimulus: barrier cue, a visual barrier blocking one of the two oblique paths; bar cue, a partially filled bar indicating the probability of the target moving in a given direction; blocked directions, target direction remained consistent within blocks of 40 trials; crash, the same as the barrier cue except that the target always crashed through the barrier. The strength of the ASEM responses was quantified as the mean ratio of horizontal eye velocity to target velocity across subjects at the onset of horizontal target motion. Error bar indicates ±1 *SE*. Adapted from Kowler et al. (2014), with permission.

Various cues can elicit ASEM responses, the strength of which depends on the cue type. The pursuit gain at the motion onset was found to be the highest when the future target direction was indicated by blocking one of two branches with a visual barrier (Figure 3). These results suggest that the visual cues depicting the physical properties of the motion path were most effective and could not be easily overridden by learning in the laboratory (e.g., repeated exposure to alternative motion trajectories through blocking motion directions).

This observation led to further investigations on the role of two-dimensional (2D) geometry in supporting predictive smooth-pursuit eye movements. Surprisingly and in contrast to what might be expected from one-dimensional (1D) pursuit, path geometry was found to play a crucial role in guiding pursuit of a target that underwent abrupt directional changes after moving for a fixed duration. Pursuit began to decelerate well before the expected time the target would change direction and reached its minimum speed just as the eye was turning (Wang, McCabe, Tournoux, & Kowler, 2019). Moreover, the magnitude of the minimum eye speed scaled with the turn angle: The larger the angle, the slower the minimum eye speed. Increasing the predictability of the trajectory of the target only reduced the time required for the eye speed to reach its minimum but did not alter the relationship between turn angle and pursuit speed.

The observed direction–speed relationship in smooth pursuit is reminiscent of the lawful relation that has been found for a wide range of biological movements (e.g., Catavitello, Ivanenko Lacquaniti, & Viviani, 2016; Huh & Sejnowski, 2016; Zago, Lacquaniti, & Gomez-Marin, 2016)—namely, the two-thirds power law (de'Sperati & Viviani,1997). The two-thirds power law states that the movement speed depends on the radius of curvature raised to the power of (1-2/3). Wang and Kowler tested whether the 2D pursuit of both predictable and unpredictable non-repetitive motions conforms to the two-thirds power law. Their findings revealed that the relationship between the radius of curvature of the eye path and eye speed resembled the predictions of the law to a considerable extent (for a comprehensive analysis, see Wang, 2022). Having established the role of geometry, specifically curvature, in 2D smooth pursuit, Kowler concluded in her recent review on predictive smooth pursuit (Kowler, Rubinstein, Santos, & Wang, 2019) that the effect of curvature points to a kinematic constraint governing natural biological motions, including smooth pursuit. Complying with such constraint, she argued, may enhance the compatibility of smooth pursuit with motions generated by other living things, thereby facilitating more precise tracking.

## Barbara Dosher's perspective

I first met Eileen in 1979 (while dealing with a mouse in her apartment!), when she was a postdoc with George Sperling at New York University. Beginning with conversations on the beach and over dinner at the Association for Research in Vision and Ophthalmology in Sarasota, we became life-long friends. We met at conferences, traveled together, and met in New Jersey or California to discuss science and life. Eileen was fascinated with the multifaceted interplay among expectations, attention, and eye movements. She and her lab produced a series of important demonstrations of “cognitive eye movements.” I was lucky enough to collaborate on a few of these, where my interests in visual attention intersected with her eye movement agenda.

Observers faced with a complex visual world process information selectively. Just as Gestalt principles influence our interpretation of spatial patterns and symmetry constrains three-dimensional (3D) shape percepts, cognitive purpose, expectations, and attention influence eye movements and how visual information is processed. To quote Eileen's 2011 review (Kowler, 2011, p. 1457): “… if there's one thing that the last 25 years has taught us, it's that eye movements are not ‘evoked’ by sensory error signals—such as motion on the retina, or a displacement of a detail some distance from the fovea. They are a response to a representation of the visual world. And, not just a representation of the objects or the visual scene, but also information about plans, goals, interests, and probable sources of rewards or useful information. Even expectations about future events.” What follows illustrates some of Eileen Kowler's influential work on the interface between cognition and eye movements, and its implications.

### Attention and smooth pursuit

One early study decisively demonstrated that selective attention mediates smooth pursuit eye movements (Kowler, Van Der Steen, Tamminga, & Collewijn, 1984). Earlier reports (e.g., Dubois & Collewijn, 1979; Mach, 1886/1906/1959; Stark, 1971) had challenged a classic view that smooth pursuit—in which a moving stimulus is tracked by the eye—was “determined solely by involuntary, automatic processing of all available stimulus motions” (Kowler et al., 1984, p. 1789). Random dot displays were cleanly equated on all other aspects of a stationary and a moving stimulus. Observers were instructed to “stay” (fixate) on the stationary dots, or to “track” the random dot motion. Eye fixation for “stay” instructions compared static alone and static plus moving dot displays, and smooth pursuit for “track” instructions compared moving alone or static plus moving dot displays. Practiced observers essentially perfectly filtered out the non-attended stimulus in a joint stationary plus moving display—as if the ignored stimulus was not present. This extraordinary demonstration revealed the strong mediating effect of attention on smooth pursuit, previously thought to be driven in whole or in part by the simple integration of motion signals. (Simple yet powerful designs such as this one are a characteristic of much of Eileen's work.)

### Attention and single saccades

Cognition and attention also share a fascinating relationship with saccadic eye movements. The ability to dissociate visual attention from the current point of fixation was observed over a century ago (Wundt, 1912). Perhaps unsurprisingly, then, early evidence regarding the connection of attention and saccadic eye movements was mixed (e.g., Klein, Kingstone, & Pontefrac, 1992; Posner, 1980; Remington, 1980). Furthermore, merely observing that eye movements often follow attention (Henderson, Pollatsek, & Rayner, 1989) fails to specify the role attention plays in saccadic control. Kowler, Anderson, Dosher, and Blaser (1995) reframed the question: Is the attention used in programming or executing a single saccade the same as the attention used in visual perception? They asked: “… whether the saccadic system ‘knows’ which is the effective target [of the eye movement] by means of the same attentional mechanism that serves perception” (Kowler et al., 1984, p. 1789). The answer to this question was yes.

**Figure 4.**
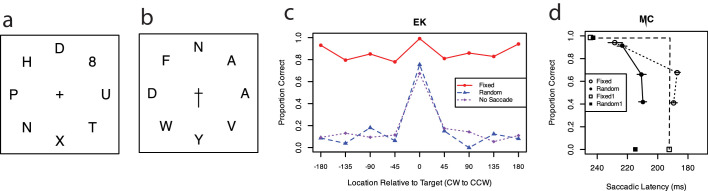
Visual attention is shifted to the goal of a single saccade. (**a**) A critical display (200 ms) was preceded and followed by a mask (500 ms), and subjects saccaded from a central fixation to (or opposite to) an attention-capturing numeral. (**b**) A letter display with an arrow cue to saccade target, with the same timing as in (**a**); subjects saccaded to the cued letter and reported a letter at a post-cued location. (**c**) Letter identification accuracy (from 10 letters) at locations relative to the simultaneously cued saccade location (at 0) revealed good letter reports only at the location of the saccade goal and poor ability to report letters at other locations (random saccade, filled circle) (subject EK). Reporting a centrally cued letter with attention alone while the eye stays at fixation is essentially the same (no saccade, triangle). (**d**) Attention-operating curve (AOC) measured by blocked instructions to favor letter identification, favor eye movement speed, or balance both. The resulting AOC (subject MC) graphs for random (filled circle) or fixed (open circle) saccade locations indicate that small increases in saccade latency are traded for relatively substantial changes in letter identification. Adapted from Kowler et al. (1995, figures 1, 5, 6, and 11, respectively), with permission.

In one demonstration, observers executed a saccade to an attention-capturing numeral singleton in an annular letter display, or they executed a saccade to the letter opposite it (Figure 4a). Saccades to the numeral had shorter latencies (by about 50 ms), more precise landing positions (by about 5° to 7° angular error), and fewer gross localization errors (by up to 12%) than saccades to the letter opposite the numeral, showing significant benefits when attention is drawn to the goal of the saccade.

In a related study, successful letter report occurred at the goal of the saccade (Kowler et al., 1995). On each trial, a randomly assigned saccade target was cued by a central pointer in the same brief display as the letters, and a post-cue marker indicated the letter to report (Figure 4b). (Comparing accuracy for a simultaneous cue and a slightly delayed cue is one standard method of measuring spatial attention; see, for example, Lu & Dosher, 2000). Letter identification is high when the letter to report and the goal of the saccade coincide (0° on the abscissa) and is essentially at chance for other locations (Figure 4c, filled circles). When the simultaneous central cue shifted attention but not the eye to the cued location (no saccade, small triangles), the pattern of letter report was virtually identical! Preparing to saccade to a location has the same effect on letter report as moving attention but not the eye to that location. Given practice and foreknowledge of a fixed location of the saccade (open circles), observers could both report the cued letter and execute the saccade—albeit with slightly delayed saccade latency and increased error. A slight delay in saccade latency and precision was traded for attention to pick up the letter identity at another location. In another study, observers were instructed to emphasize the saccade or the letter report or to balance the two to measure the attention operating curve (Navon & Gopher, 1979; Sperling & Dosher, 1989; Sperling & Melchner, 1978). Observers voluntarily traded relatively small delays in saccadic latency for significant improvements in letter report (Figure 4d). Similar findings—namely, high target identification when the letter coincided with the goal of a saccade—have been reported by others (Deubel & Schneider, 1996; Hoffman & Subramaniam, 1995). Attention to the target of the next saccade may even be important in picking up small details for microsaccades (Kowler, 2024).

### Attention in saccade sequences

**Figure 5.**
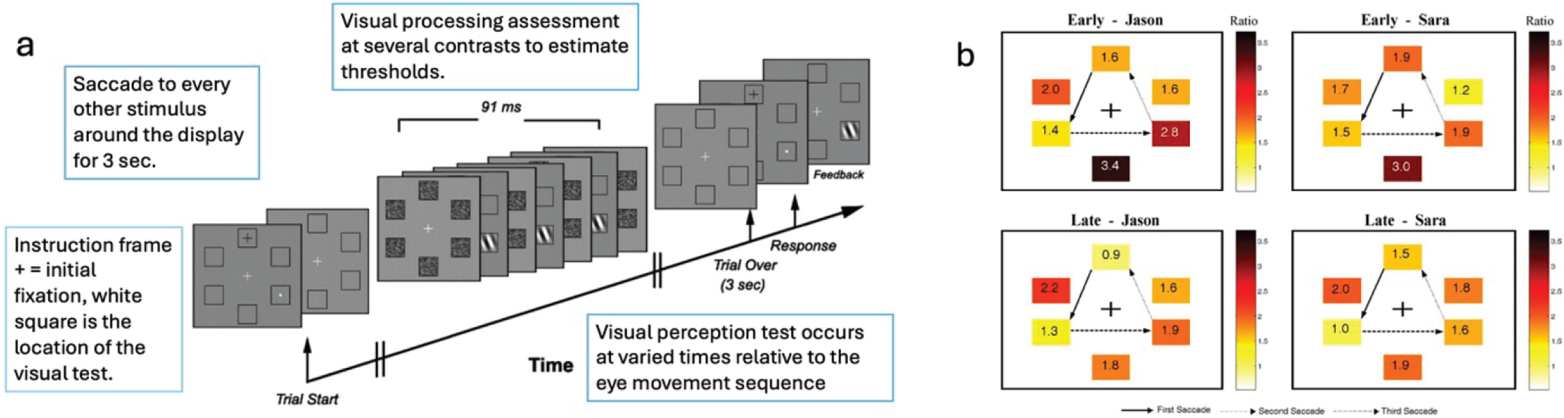
Perceptual attention selects the spatial location of the next saccade goal in a saccadic sequence. (**a**) Subjects executed saccades to every other box around the circle for 3 seconds, with starting fixation at the + marked location (varied over trials) and attention cue at the white square. A masked visual test (oriented patch top left or right) occurred at various locations during an intersaccadic pause either earlier or later in the saccade sequence. (**b**) The ratio of contrast thresholds during the saccade sequence relative to contrast threshold during stable fixation (retinal eccentricity control; current fixation location in saccade sequence is rotated to the top in the graph). Direction of the saccade sequence (counterclockwise here) is indicated by the arrows. Threshold ratios are best (lowest) at the goal of the upcoming saccade, especially compared with the eccentricity control (opposite location relative to current fixation at the top), followed by current fixation, with the worst perception at the location of highest eccentricity and off the saccadic path (bottom). Adapted from Gersch et al. (2004, figures 1a and 5b), with permission.

Most naturalistic situations involve sequences of saccades. Kowler and colleagues extended the investigation of how visual attention is related to saccades to saccade sequences or in more complex tasks. Visual target identification was measured during eye movement sequences at various locations relative to current fixation. One experiment examined a stereotyped sequence of saccades to every other location around an annulus (Figure 5) (Gersch, Kowler, & Dosher, 2004). Visual tests patches were masked because spatially cued attention is most robust when masked (Dosher & Lu, 2000). Thresholds during saccade sequences were generally higher and were best at the location of the next saccadic goal. There was no evidence of a spread of attention further along the sequence beyond the immediate next saccade. Here, too, the attention used in saccade programming was the same attention that subserved perception.

**Figure 6.**
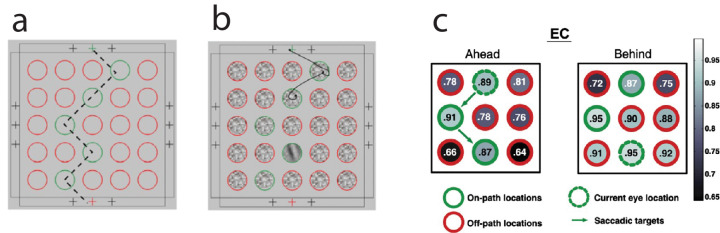
Perceptual attention assessed during a sequence of eye movements guided by the green visual cues. (**a**) The sequence of eye movements on one trial, from top to bottom, are indicated by the green circles. (**b**) A display for visual test (moderate contrast oriented patch) during an intersaccadic pause, here after the second saccade. (**c**) Visual discrimination accuracy in the central nine locations when the path is ahead of the current eye location or behind the current eye location. Visual discrimination, associated with attention, is deployed along the path of the eye. Adapted from Gersch et al. (2009, figures 1 and 3), with permission.

In contrast, when saccades follow a visually marked path, perceptual identification is best along the saccadic path (Figure 6) (Gersch, Kowler, Schnitzer, & Dosher, 2009). Visual attention was most focused on the next saccade goal, but there was also a benefit of feature attention (to color) along the marked path. Feature attention operates more widely across the visual field (Cohen & Maunsell, 2011; Liu, 2019; Martinez-Trujillo & Treue, 2004; Maunsell & Treue, 2006; Theeuwes, 2013). The conclusion was that “… attention could be allocated beyond the target of the upcoming saccade to other locations along the saccadic path provided … [it] was marked by a perceptual cue” (Gersch et al., 2009, p. 1264).

### Complex tasks and open questions

Kowler's studies on the interplay among attention, perception, and eye movements were influential in shifting the field toward seeing eye movement control in a system that interacts with complex task demands. Visual attention has a key role in the control of saccades, and the connection between visual identification and saccades depends on the demands of the cognitive task motivating the eye movements and the demands of the visual test. For example, more demanding counting tasks limit visual identification more than pointing or just looking, whereas less demanding visual tasks such as localization are less sensitive to cognitive task demands (Wilder, Kowler, Schnitzer, Gersch, & Dosher, 2009). On the other hand, visual attention and saccade timing and localization can be more diffusely connected in situations in which a distractor is physically close to the intended saccade target and affects fixation location (Wollenberg, Hanning, & Deubel, 2020). Or, situations in which visual information drives a competitive saccade decision may involve preparation for two saccades (e.g., Jonikaitis, Klapetek, & Deubel, 2017).

An open active question is exactly how the brain implements shifts in attention and programming of eye movements within a complex visual field. Some early physiological reports emphasized the potential decoupling of attention and saccadic planning in the frontal eye field (Bisley & Goldberg, 2003; Murthy, Thompson, & Schall, 2001), suggesting that it may code the location of attention even when a saccade is withheld or suppressed. The question is how the interplay between perceptual attention to the next saccadic target and other forms of spatial attention helps coordinate integrated perception through sequences of eye movements (Jonikaitis, Szinte, Rolfs, & Cavanaugh, 2013; White, Rolfs, & Carrasco, 2013).

## Zygmunt Pizlo's perspective

Pizlo, during the last 40 years, expanded his original interest in symmetry that motivated his poster at ECEM 3. He found the role of symmetry not only in 3D visual perception (Dickinson & Pizlo, 2013; Pizlo, 2008; Pizlo, Li, Sawada, & Steinman, 2014), but also in human problem solving and scientific discovery (Pizlo, 2022). Being a member of the Kowler and Steinman group, Pizlo was also involved in studying head–eye coordination under natural viewing conditions. This involved being actually “pushed around” by Han Collewijn (Epelboim et al., 1995). Collewijn made our work possible through his help in designing the Maryland Revolving Field Monitor^1^ and developing silicon annulus-sensor coils for eye-movement recording.

### The playbill

There has been considerable progress in theoretical vision science since our group was formed in 1985, particularly in 3D shape perception, the simplicity (least-action) principle, and inverse (inference) problems. These and many other concepts were put forth as our historical analysis progressed from Ernst Mach and Gustav Fechner, Max Wertheimer, Ernst Cassirer, Roger Shepard, David Marr, David Foster, Horace Barlow, Tomaso Poggio, and Irving Biederman.^2^ The sections that follow explain things we understand now but did not in 1985. The bottom line will turn out to be that theories developed from within the nativistic approach and based on a simplicity principle have fared better than those developed from the empiristic approach so popular these days.

### The origin of main concepts

What follows below is based on Pizlo's work after he joined the University of Maryland (1988–1991), which is when our group story begins, followed by his 26-year work at Purdue University (1991–2017) and finally, at the University of California, Irvine. Psychophysics was introduced in the 19th century by two physicists: Gustav Fechner (Fechner, 1860/1966) and Ernst Mach (Mach, 1886/1906/1959). Fechner focused on experimental methodology, and Mach provided a solid background for formulating theories.^3^ Mach, being almost four decades younger than Fechner, benefited from modern developments in mathematics and physics—namely, symmetry and the least-action principle. Fechner did not make use of either of them. First, recall that symmetry was established as the foundation of geometry by Felix Klein in 1872, which was a dozen years after Fechner published his book titled *Elements of Psychophysics*. Mach published his psychophysics book in 1886, and he knew about Klein's reformulation of geometry based on the concept of symmetry. Second, there is no evidence in Fechner's writings that he was aware of the role of a least-action principle in physics, whereas Mach wrote a book in 1883 promoting the least-action principle as the foundation of physics. Quite possibly soap films provide the simplest and most intuitive illustration of a least-action principle. Local forces of attraction among soap molecules result in soap films producing minimal surfaces, which are surfaces of minimal area (Hildebrandt & Tromba, 1996). When you dip a wire rectangle in soap water and you take it out, the soap film will form a planar surface which is a surface of minimal area considering the boundary. Now put a thread forming a loop on the surface of this soap film. At this point, nothing interesting happens. But, after you perforate the soap film inside the loop, the thread will take the shape of a circle (watch the movie at https://www.youtube.com/watch?v=v-R1mJ2gKDw). Why a circle? The soap film minimizes the surface area outside the loop. This maximizes the surface area inside the loop. It is known that from all closed curves having a fixed perimeter, a circle has maximal surface area. Soap film solved the problem in the calculus of variations, the branch of calculus developed by Euler and Lagrange in the 18th century (for a lucid overview, see Hildebrandt & Tromba, 1996). This result is quite remarkable, but we must remember that physical systems, such as soap films, electromagnetic radiation, or mechanical systems, rely exclusively on spatially local interactions. Physics does not use action at a distance. We will get back to this aspect of the least-action principle at the end of our paper.

These two concepts, symmetry and least action, shaped the scientific approach of Mach, who viewed perception as an inference governed by the simplicity principle (a form of a least-action principle). It is in this way that Mach was a true precursor of Gestalt psychology. In contrast, Fechner viewed perception as a psychological measurement. Although Fechner's approach provided the foundation for some aspects of psychophysics that played an important role in the 1960s, it fell out of the mainstream of modern research because it could not incorporate a formalism central to the inverse problems theory (Pizlo, 2001).

**Figure 7.**
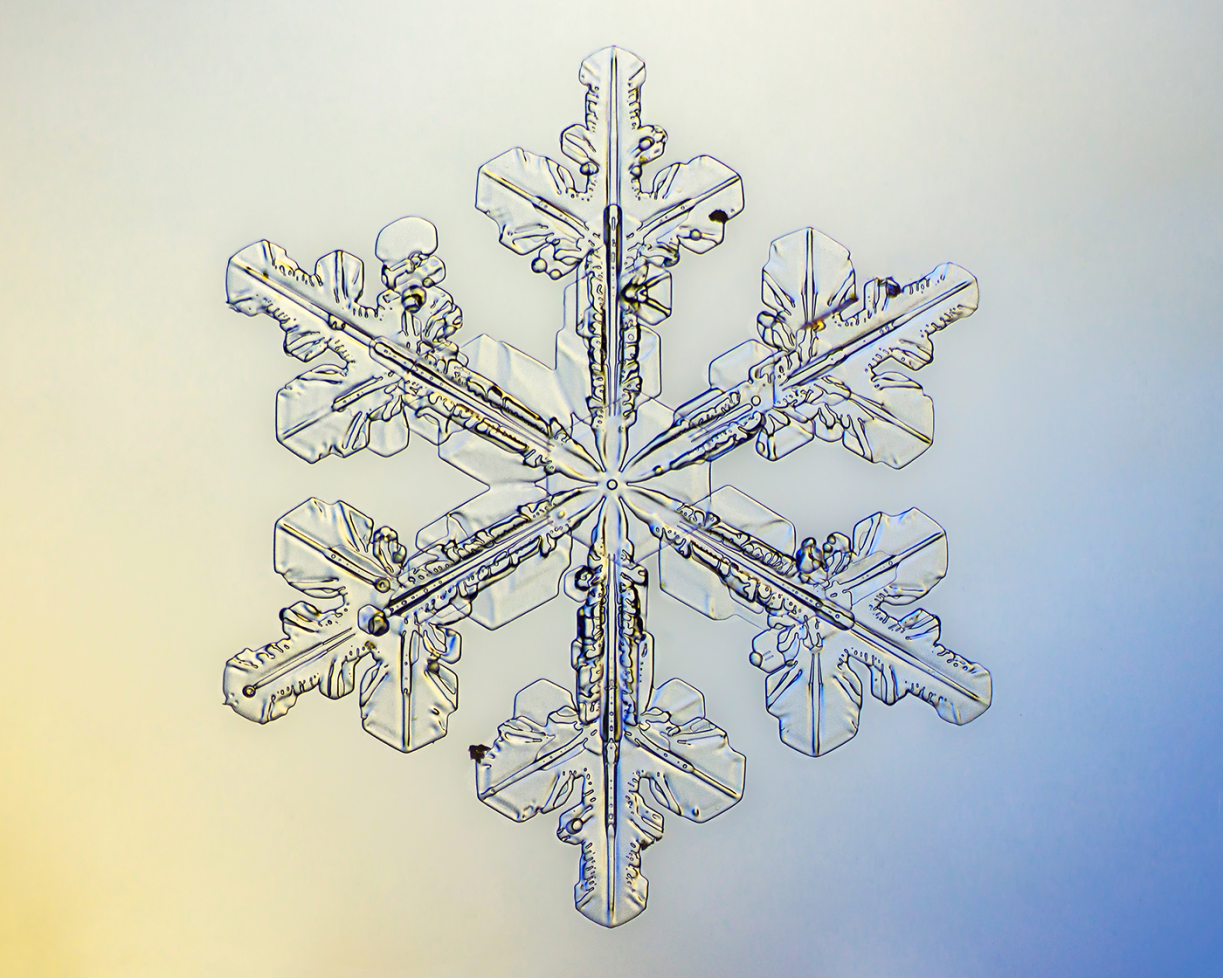
Snowflakes are invariant to rotations. They are also characterized by redundancy. Image by Janek Lass (https://commons.wikimedia.org/w/index.php?curid=145499964).

So, how is symmetry defined in math and physics? Richard Feynman, a Nobel Prize winner in physics, stated (after Hermann Weyl) that “a thing is symmetrical if there is something we can do to it so that after we have done it, it looks the same as it did before.” Natural laws are the same across many different conditions (translation in space or time). This invariance is what is referred to as the symmetry of the laws of physics (Rosen, 2008). This is completely analogous to how mathematicians define symmetry. Klein (1872) showed that rigid motion (plus size scaling) defines Euclidean geometry.^4^ Klein referred to Euclidean geometry as a symmetry group because shapes of figures and objects do not change after rigid motion and size scaling. Snowflakes are often used to illustrate the concept of symmetry (see Figure 7). Indeed, when a snowflake is rotated by a multiple 60°, its shape does not change nor its position. If you did not look at it while I was rotating a snowflake, you would not know whether any transformation had been applied. If a snowflake is rotated by another angle and/or if it is translated, you would know that a transformation was applied. In this case, the position of the snowflake changed, but not its shape or size.

Mach, in his psychophysics book published in 1886 (Mach, 1886/1906/1959), formulated what was later referred to as a transposition principle, and he illustrated it with examples taken from vision and audition. In vision, he pointed out that it is natural to see two triangles as having the same shape when one is a scaled version of the other. By a scaled version he meant that one figure is produced by multiplying all sides of the triangle by the same scaling factor. Uniform size scaling is just one element of Klein's Euclidean geometry. The other elements of Klein's Euclidean geometry are rigid translation, rigid rotation, and mirror reflection. Mach illustrated all four transformations in the same place in his psychophysics book (Mach, 1886/1906/1959, pp. 107–108). These four transformations form a symmetry group that was described by Klein in his 1872 *Erlanger Programm*. In auditory perception, Mach pointed out that a melody is perceived as the same after we transpose the melody to a different key.

Soon after Mach published his psychophysics book, Von Ehrenfels (1890) made a big impact by using Mach's ideas to introduce the concept of Gestalt qualities and laying the foundations for the Gestalt school of psychology. Von Ehrenfels gave full credit to Mach for introducing the concept of invariance under transformations in visual and auditory perception. Von Ehrenfels termed this invariance a transposition principle and the resulting percept a Gestalt quality.

Gestalt psychology was established by Wertheimer, Koffka, and Köhler in the beginning of the 20th century not long after Von Ehrenfels’ publication.^5^ The birthdate of Gestalt psychology as a formal school has often been stated as 1912, which is a reference to a paper by Max Wertheimer in which he described a pure motion he referred to as the Phi phenomenon (see Steinman, Pizlo, & Pizlo, 2000). Using motion to start a revolution in psychology was quite fitting, considering that motion was used to reformulate Euclidean geometry (Klein, 1872). For Klein, rigid motion allowed bringing symmetry to geometry. Whether or not Wertheimer also thought about symmetry in 1912 is not clear, but 11 years later he was already using symmetry as one of the most important concepts in visual perception (Wertheimer, 1923). Gestalt psychologists cited Von Ehrenfels but not Mach or Klein. Ignoring Klein might have been natural, as Klein was not studying perception, even though Klein pointed out on several occasions that geometry is a formalization of visual perception and that it would have been impossible to talk about geometry without any reference to perception (e.g., Klein, 1925/1939, p. 187). But, ignoring Mach's psychophysics is more difficult to justify, considering his ideas, examples, and formalism, as well as his impact on Von Ehrenfels’ writings. Koffka (1935) commented on these omissions (on p. 63), where he pointed out that the philosophical motivation of Mach's (1886/1906/1959) psychophysics made it difficult to incorporate his ideas into Gestalt psychology. Today, we are less bothered by Mach's philosophy. Mach's thinking represented *logical positivism*, which emphasized the role of theories in *describing* but not *explaining* nature. This approach was useful when Mach justified a least-action principle and preferred this principle over Newton's physics. Mach suggested that a least-action principle could simply be the result of applying the economy of thought in formulating descriptions of nature. According to Mach, we will never know how nature works, so scientists should focus on providing the simplest and most intuitive description of natural phenomena. This logic allowed Mach to sidestep the question of the teleological aspects of a least-action principle. According to a least-action principle, nature solves an optimization problem and chooses the least-cost (cheapest, simplest) solution for achieving the final state. As an example, when you throw an object in the air, the motion of the object follows a parabolic path not because of Newton's second law of motion described by the differential equation but because the parabolic path minimizes action, which is defined as the time integral of kinetic energy minus potential energy between the starting point and the endpoint. Newton did not have to know what the endpoint of motion was. The endpoint of motion was determined by succession of causal chain of events described by his second law of motion. However, a least-action principle assumes that the endpoint is known before the movement begins. It is easy to see why many physicists were uncomfortable with a least-action principle starting in 1662 when Fermat proposed its first version. And, they continued to be bothered, all the way until 1948 when Richard Feynman provided a solid justification of a least-action principle in quantum physics by introducing the concept of path integral. Similarly, as light rays interfere, so do particles, leading to destructive interference. As a result of this interference, only one path survives, and this path represents the minimum of action. This is analogous to least-time principle of Fermat on one side and Huygens's wavefronts, on the other.

### Two aspects of symmetry^6^

Now, let's explore another aspect of symmetry, its redundancy. A snowflake consists of six copies of one section (see Figure 7). This self-similarity (self-identity) results in redundancy. The mirror symmetry of animal bodies refers to self-similarity, too. As a result, mirror-symmetrical objects are redundant, too, because one half is repeated twice. So, symmetry has two related aspects. The first, described in the previous section, is invariance under transformation. When a physical law (say, Newton's second law of motion) applies to different spatial positions (e.g., an experiment performed in two labs) and to different times (e.g., an experiment performed today and tomorrow), we say that the law is invariant. This is referred to as the symmetry of the law. The same can be said when an object is moved rigidly in physical space. The object does not change (it is invariant). This is referred to as symmetry in the presence of a rigid motion. The second aspect, redundancy, is different. When an object is self-similar, we say that the object is symmetrical: A 3D reflection of a mirror-symmetrical object results in the same object. These two aspects of symmetry are described by the same mathematics, so they are closely related. But, in one case, the symmetry is in the transformation, whereas, in the other, the symmetry is in the object. These two aspects of symmetry—invariance and redundancy—play different roles in visual perception, but this fact has been overlooked by vision scientists. This is explained next.

**Figure 8.**
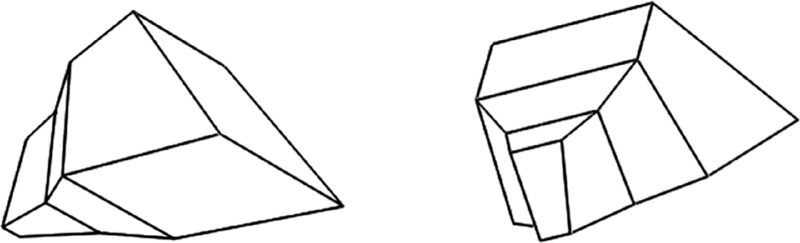
These two mirror-symmetrical shapes are identical. This figure illustrates the concept of shape constancy, which is the 3D invariance of the percept in the presence of a rigid rotation.

Perceptual constancy refers to the invariance of a percept in the presence of a transformation in the viewing conditions. Take shape constancy. When you look at a given 3D object from two different viewing directions and you see it as having the same shape, you have achieved shape constancy (see Figure 8). Perceptual constancy fits the definition of symmetry as an invariant of the transformation group (Cassirer, 1944). In the case of shape constancy, a 3D rotation is the relevant transformation group, and the symmetry is in the transformation. But, for shape constancy to be achieved, the 3D shape must first be reconstructed from a 2D image. This reconstruction goes beyond the invariance aspect of symmetry. Reconstructing a 3D shape from its 2D image is an ill-posed problem because a single 2D retinal image is consistent with infinitely many 3D configurations (Pizlo, 2001). How does the visual system choose one 3D interpretation out of the infinitely many possible? Redundancy in (the symmetry of) the 3D shape makes the problem well posed (Pizlo et al., 2014; Pizlo & de Barros, 2021). It has been established that a single 2D perspective image of a mirror-symmetrical 3D shape determines this 3D shape uniquely, and a single 2D orthographic image of a mirror-symmetrical 3D shape determines this 3D shape up to one free parameter, the aspect ratio (Sawada, Li, & Pizlo, 2011). The uniqueness of the reconstruction of a mirror-symmetrical shape from a 2D orthographic image is achieved by maximizing 3D compactness.

When symmetry is in the transformation, we refer to it as a Gestalt quality, following Von Ehrenfels’ terminology. It results from the transposition principle, which is Von Ehrenfels’ name for an invariance under a transformation. It follows that we can classify 3D shape constancy as a Gestalt quality. What can we say about the reconstruction of a 3D shape from a single 2D image? Depth is lost in the projection from 3D to 2D. In order to reconstruct the 3D shape, information must be added to the 2D image. The only way to do it is to use the redundancy aspect of the symmetry of the object (see the previous paragraph). It seems to us that what Gestalt psychologists refer to as emergent properties in perception (or simply Gestalt) refers primarily to cases in which a 3D shape is produced from a 2D image.^7^ The 3D percept in such cases cannot possibly be explained by an analysis of the 2D image. This phenomenon is captured by the often-quoted statement that reads: “The whole is different from the sum of the parts.” Look at the animation in this link: https://sites.socsci.uci.edu/∼zpizlo/GestaltCube. A regular hexagon is stationary, whereas the Y junction is jumping around. Nothing else is there. Just these two 2D patterns. When you see a rigid Y junction moving around, you experience a Gestalt quality, which is an invariance (transposition principle) of the percept under transformations. When you suddenly see a 3D cube, you experience an emergent property, or a Gestalt. The 3D shape percept is different from the sum of 2D retinal patterns. We will say more about emergent properties next.^8^

### Examples of the redundancy aspect of symmetry in 3D vision

Consider two classical studies that started 3D object perception research at the time when the cognitive revolution was about to begin in 1956. We are referring here to Hochberg and McAlister (1953) and Wallach and O'Connell (1953). These two papers appeared in the same journal, in the same year, in successive issues. The former illustrated the operation of a simplicity principle rooted in the nativism of Gestalt psychology, whereas the latter suggested the role of experience rooted in empiricism. As such, these two papers seemed to represent two opposite views of how 3D vision works, but this traditional understanding looks very different when we bring the redundancy aspect of symmetry into the analysis. Hochberg and McAlister had some intuition about this, whereas Wallach and O'Connell were unaware of it. Note that it is important to appreciate the significance of both contributions.

**Figure 9.**
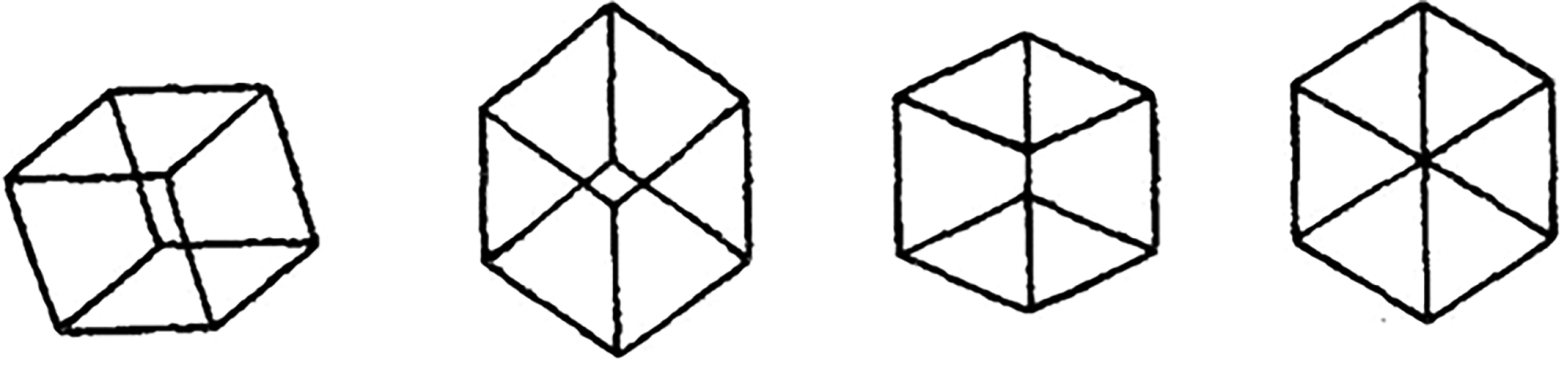
Stimuli used by Kopfermann (1930) and by Hochberg and McAlister (1953). These are four projections of a Necker cube. From Kopfermann (1930), with permission from Springer Nature.

Take the study by Hochberg and McAlister first. They used four orthographic images (line drawings) of a transparent cube (see Figure 9). Two of these images were easily perceived as 3D shapes, and the other two were perceived as 2D patterns. The critical factor was the degree of symmetry of the 2D image. The more symmetrical the 2D image was, the less likely the 3D percept. The 3D interpretation, a cube, was the same for all four images, so the degree of 3D symmetry was the same for all four images. The authors were hoping that the best explanation would be based on information theory, which is based on probability theory. Information theory offers a principled answer to the question of establishing the minimal amount of information necessary to describe the stimulus and the percept. This was an interesting and influential idea in its day. If a stimulus or a percept can be compressed, it means that it has redundant information (is self-similar). Back in 1953, such tools did not exist, so Hochberg and McAlister could not have provided a formal theory.

Next, let's describe what Wallach and O'Connell (1953) did. Hans Wallach was trained by Wolfgang Köhler, one of the three founders of Gestalt psychology. As a result of this training, Wallach started his research assuming that the visual system is equipped with innate perceptual mechanisms that are responsible for forming percepts, especially three-dimensional percepts. Over time, however, Wallach found himself incorporating empiristic elements into his experiments and thinking. His 1953 paper with O'Connell stated the following as the motivation of their study: “Unfortunately it appears that no one has succeeded in formulating rules of spontaneous organization adequate to predict which pattern of retinal stimulation will lead to perceived flat figures and which one will produce three-dimensional forms” (pp. 205–206). They pointed out that a 3D percept can be formed easily as a result of looking at the shadow of a rotating rigid object, even if the three-dimensionality of the object is not conveyed by any pictorial cues. It was natural for Wallach to assume that motion could be critical in the early visual experience of humans. He further assumed that the use of motion for seeing 3D objects could be learned and transferred to novel objects. We now know that learning is not involved and that the visual system always applies a 3D rigidity constraint when multiple 2D images of an object are shown to the observer. Rigidity can be applied because rigidity provides the necessary redundancy. The observer has multiple 2D images of the same 3D object (see an example of a kinetic depth effect, also known as structure from motion, in this link: https://michaelbach.de/ot/mot-sfm/). The 2D motion on the computer screen is not rigid, but there is a 3D rigid interpretation. Here, redundancy (symmetry) is in the 3D transformation, not in the object. Recall that rigid motion is the foundation of Euclidean geometry according to Klein (1939). Rigid motion includes rotation, translation, and mirror reflection. All three are considered symmetries. So, these two papers published in 1953 illustrated the operation of two different subgroups of Euclidean symmetry group: 3D mirror reflection and 3D rotation. No wonder that both are effective in producing a 3D percept from one or more 2D images. However, the perception of mirror-symmetrical shapes is much closer to veridical than the perception of non-symmetrical shapes from motion.

To summarize, the invariance aspect of symmetry could be present in the transformation (e.g., rigid motion of an object keeps the object the same) or in the object itself (3D reflection of a mirror-symmetrical object results in the same object). Same with the redundancy aspect of symmetry. Redundancy could be present in multiple views of a rotating rigid object or in the self-identity or self-similarity of a mirror-symmetrical object (the two halves are identical or similar). Both forms of redundancy are essential in 3D reconstruction from 2D images. As pointed out above, depth is lost in the projection from 3D to 2D, and information must be added to the 2D image. The only way to do it is to use redundancy (symmetry): redundancy in the object (e.g., mirror-symmetrical objects) or redundancy in the transformation (multiple views in structure from motion and in binocular vision).

It is obvious that producing a 3D representation of shapes and scenes goes beyond Mach's and Von Ehrenfels’ transposition principle. The 3D representation is produced from one or more 2D images, and this representation is often veridical or nearly so. By veridical we mean that we see things the way they are “out there.” The fact that the 3D shape of an object is invariant in the transformation from the physical world to the mental world is a form of conservation, like conservations in physics. When I look at a wooden chair, my percept is not made of wood, and it does not have weight. So, the material from which the chair was made and its weight are lost in the transformation from the physical to the mental, but the perceived shape is identical (or nearly so) to the shape of the chair. 3D shape is conserved in the transformation from physical to mental. This is analogous to conservations in the physical world. When two cars collide, the cars are destroyed, but not everything has changed. The total linear momentum is conserved. It turns out that the mathematical formalism behind 3D shape reconstruction (as a Gestalt or as an emergent property) is very similar to the formalism behind physical conservations. The emergent property of conservation laws in physics was established by Emmy Noether (Noether, 1918) when she proved a theorem showing how conservation laws are implied by the symmetry of nature and a least-action principle.^9^ Gestalt psychologists might have been aware of the similarity among symmetry, least-action, and conservation laws in physics, on the one hand, and symmetry, simplicity, and veridicality in perception, on the other; however, if they were aware, they did not elaborate on it. Köhler did speculate about the role of physics in the isomorphism between the brain and a percept (Köhler, 1920), but he never asked questions about conservations. Neither did Wertheimer, but Wertheimer could have been aware of this through his conversations with Einstein (Wertheimer, 1945). It was Einstein who introduced symmetry as the essential foundation of physics, and he was aware of Noether's celebrated theorem because he needed her theorem to publish the general relativity theory.

## Epilogue

It is obvious to us that the Gestalt revolution in the beginning of the 20th century did not happen in an intellectual vacuum. The Gestalt revolution was a part of a larger revolution that happened across three disciplines: mathematics, physics, and psychology. Felix Klein's introduction of symmetry to geometry, Mach's use of symmetry and a least-action principle in physics, Einstein's claim that a symmetry assumption takes precedence in theory formulation over experimental results, and Noether's derivation of the conservation laws from symmetry provided the mathematical foundation for Gestalt ideas. This mathematical context was there when Gestalt psychology began to emerge, and it seems very unlikely that these early Gestalt psychologists completely missed it. Invariance in nature was known to Mach, and it led to Von Ehrenfels’ Gestalt qualities. The simplicity principle (aka Prägnanz) was an analog of a least-action principle, and conservation laws found their counterpart in the concept of the Gestalt as an emergent property. If Gestalt psychologists were aware of this context, it is unfortunate that they did not point this out in their writings. Had they done so, their revolution would have been received more seriously and would not have been abandoned prematurely, as it was.

As a final note, the Gestalt revolution benefited from the revolutions in mathematics and physics, but it also laid foundations for new directions in the natural and engineering sciences. The way Gestalt psychologists described perception made it natural to adopt the inverse problems approach to perception and beyond. The first systematic treatment of inverse problems as a new class of problems was presented in the 1960s by Tikhonov and his group (Tikhonov & Arsenin, 1977), but it was not formally recognized by vision science until 1985 (Poggio, Torre, & Koch, 1985). Interestingly, the regularization methods for solving inverse problems are mathematically very similar to variational methods used in the least-action principle in physics. Mach understood this connection (Mach, 1883/1919; Mach, 1886/1906/1959), which can be seen in his frequent comparisons between the least-action principle in physics and the simplicity (economy of thought) principle in perception. Mach had a full grasp of the existing formalisms in physics but could not translate them to perception. It was not until almost a century later that Foster (1978) showed how this works in the case of apparent motion. One way to see how the regularization theory of solving inverse problems in vision generalizes the least-action principle known in physics is to realize that, although soap film can produce a perfect circle, as described above, it will never produce the shape of a butterfly or any other mirror symmetrical figure. The main reason is that mirror symmetry requires spatially global computations, whereas physics always uses spatially local interactions. In physics, there is no action at a distance. In perception, there is. One may only wonder how our field of vision science would have fared if Gestalt psychologists had taken Mach's ideas more seriously and had reached out to mathematicians and physicists of that period with an explicit plan of providing formal foundations for their revolution. As mentioned above, Wertheimer did talk to Einstein about symmetry in science. Von Ehrenfels learned about symmetry and invariance in perception from Mach, and Köhler took classes from Max Planck. But, apparently these interactions were not sufficient. The formal key to the concept of Gestalt as an emergent property could have been derived from Noether's theorem in which she showed how conservations result from symmetries by the application of a least-action principle (Noether, 1918), but Gestalt psychologists missed Noether's contribution. This was not surprising because even contemporary physicists tended to downplay the fundamental discovery of a female colleague. Conservation principles have begun to play a role in cognitive psychology relatively recently. Isaac Weiss (Weiss, 1994; Weiss, 1997), who worked at the University of Maryland for years, was the first. He derived new invariants of shape from shading as conservations by applying Noether's theorem. Huh and Sejnowski (2016) derived the 2/3 power law for hand movements as conservations, also applying Noether's theorem. One of us (Wang, 2022) studied the relation between predictive eye movements and the 2/3 power law, verifying the extent to which the theory of hand movements can generalize to the motor control of smooth eye movements. Finally, another one of us (Pizlo & de Barros, 2021) showed that 3D shape perception can best be viewed as a conservation and suggested that 3D shape perception and parity of mirror-symmetrical objects are conjugate variables, in a way analogous to the conjugate variables in mechanics—namely, spatial position and linear momentum or temporal position and energy.

In conclusion, we can say that the cognitive science which was rooted in the Gestalt tradition proved to be essential in the work of our group that included and often was dominated by the ideas of Eileen Kowler. We feel that it is worthwhile to recognize the close connections to the revolutions in math and physics that happened contemporaneously with the Gestalt revolution. We hope that this note does justice to the impact of Kowler's work as well as to the theoretical musings of our group.

## Footnotes

1 The documentation of the instrument can be found in Edwards et al. (1998).

2 The anonymous reviewer encouraged us to add names of Gibson and Koenderink to our list. Gibson (1950); Gibson (1979) did emphasize the importance of studying 3D vision, but he focused on the possible role of projective invariance in perception of surfaces, rather than shapes. Furthermore, he excluded constraints in 3D vision, which was a major mistake in our view. Koenderink (1990) also focused on invariants of the projection from 3D to 2D and did not include constraints in his theories of vision. We did not find either Bayesian or regularization models in Koenderink's papers.

3 We view Mach's psychophysics primarily as a theoretical rather than experimental contribution. This can be seen by the fact that he discussed symmetry in physics and perception, as well as the least-action (aka simplicity or Prägnanz) principle in physics and perception.

4
Klein (1925/1939) explained how a rigid motion group (plus size scaling), and the corresponding invariants provides a complete and coherent description of Euclidean geometry and avoids all the difficulties of the version presented by Euclid in his *Elements*.

5 Von Ehrenfels represented the Graz school of Gestalt psychology that was established by Meinong.

6 An anonymous reviewer pointed out that there is large literature on symmetry, which is true. This paper focuses on 3D symmetry and its application as a constraint in solving an ill-posed inverse problem of 3D shape reconstruction. This application of symmetry received much less attention in prior research.

7 Gestalt psychologists often used 2D examples to illustrate Gestalt as emergent properties in perception. Check, for example, Figure 13 in Wertheimer (1923) where he showed a superimposed sine wave and square wave. The ambiguity was at the *X* intersections, and Wertheimer pointed out that the regularity (symmetry) of the periodic patterns removes the uncertainty. The symmetry is the source of redundancy that allows solving an ill-posed inverse problem in this case. The same is true with most, if not all grouping principles, by proximity, similarity, good continuation, common fate, etc. Grouping is ambiguous unless the visual system identifies a constraint representing local redundancy that leads to a unique interpretation.

8 We are effectively offering English labels for the confusing and ill-defined German terms “Prägnanz” and Gestalt. We realize that not all readers will agree with our proposal. The Prägnanz principle is a simplicity principle in perception, and it is derived from the concept of a least-action principle in physics. So, instead of saying “Prägnanz” one should say “simplicity.” This was simple. Gestalt, on the other hand refers to two concepts. Gestalt quality as defined by Von Ehrenfels means invariant of a transformation. This is illustrated by the invariance of melody when we transform music from one key to another, or the invariance of shape when the object undergoes rigid motion. So, instead of saying “Gestalt quality,” one should say “invariance.” Gestalt, as an emergent property, refers to what physicists refer to as conservation. In perception, it always represents the result of an inference where constraints are combined with the sensory input. So, instead of Gestalt one should say “psychophysical conservation” or “psychophysical inference.” In these cases, the percept cannot be decomposed into sensory parts. For example, the 3D shape percept is not a sum of percepts of 2D features on the retina.

9 See Tavel (1971) for the English translation of Noether's paper.
